# Multi-scale modelling with spikes and rate codes: a demonstration in a model of the basal ganglia

**DOI:** 10.1186/1471-2202-14-S1-P154

**Published:** 2013-07-08

**Authors:** Alexander Blenkinsop, Kevin Gurney

**Affiliations:** 1 Department of Automatic Control & Systems Engineering, University of Sheffield, South Yorkshire, S1 3JD, UK; 2 Department of Psychology, University of Sheffield, South Yorkshire, S10 2TP, UK

## 

Computational models of neural systems exist at varying levels of description, from detailed models of individual neurons to 'mean field' or 'neural mass' models which represent neural populations. Within this spectrum, there are two broad classes: those that explicitly use discrete spikes, and those which use continuous scalar values to represent average firing rates. Within each class, disparate levels of description can co-exist; for example, in combining detailed conductance-based neurons and leaky-integrator neurons. However, there is a schism in signal representation between spiking and rate-coded models which means they are ostensibly incompatible with each other. Nevertheless, if we wish to understand how changes in cellular or microcircuit structure impact on the brain at a *systems *or behavioural level, then the ability to robustly unify models across this divide ('spikes and rates') will provide an invaluable tool, since systems models are often more appropriately built using rate-coded components. To address this, we developed a novel hybrid-modelling methodology in which the membrane potential of a spiking neuron is converted to an instantaneous measure of firing rate. This measure is based on the value of a 'conversion function' which is incremented by a fixed amount if the neuron fires, and decays exponentially otherwise.

The vehicle for investigating the hybrid approach was an existing rate-coded model of the basal ganglia whose properties are well-understood [3]. We replaced the leaky-integrator neurons in the striatum with Izhikevich neurons [1], modulated for the effects of dopamine [2]. The striatum is a useful target for more detailed modelling as it has the highest neuron count in the basal ganglia, and comprises a complex microcircuit with several populations of interneurons. The parameters of the conversion function were calibrated according to firing rate data from [2]. We also developed a method for obtaining the minimal number of spiking neurons to reliably represent a single leaky integrator. The resulting firing rate output from the striatum was then fed into a rate-coded model of the rest of basal ganglia [3], parameterised to yield physiologically plausible ranges of firing rates.

The performance of the hybrid model was tested against its parent models and found to replicate the behaviour of the latter over the full range of possible inputs. Further, as a first demonstration of the ramifications of a phenomenon grounded in spikes, at the systems level, we used spike-timing dependent plasticity at cortico-striatal synapses to implement learning in basal ganglia.
